# Biocompatible Upconverting Nanoprobes for Dual-Modal Imaging and Temperature Sensing

**DOI:** 10.1021/acsanm.3c06111

**Published:** 2024-03-05

**Authors:** Egle Ezerskyte, Augustas Morkvenas, Jonas Venius, Simas Sakirzanovas, Vitalijus Karabanovas, Arturas Katelnikovas, Vaidas Klimkevicius

**Affiliations:** † Institute of Chemistry, Faculty of Chemistry and Geosciences, Vilnius University, Naugarduko 24, LT-03225 Vilnius, Lithuania; ‡ Biomedical Physics Laboratory, National Cancer Institute, Baublio 3b, LT-08406 Vilnius, Lithuania; § Department of Chemistry and Bioengineering, Vilnius Gediminas Technical University, Sauletekio 11, LT-10223 Vilnius, Lithuania

**Keywords:** upconverting nanoparticles, nanothermometry, MRI contrast, nontoxic, stable aqueous colloids

## Abstract

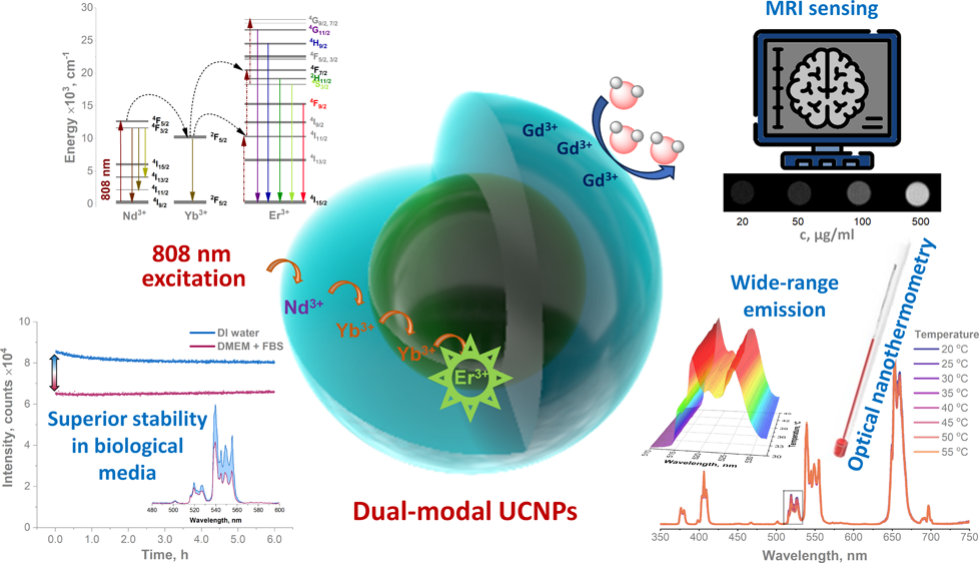

The demand for multimodal nanomaterials has intensified in recent years driven by the need for ultrasensitive bioimaging probes and accurate temperature monitoring in biological objects. Among the different multimodal nanomaterials that have been extensively studied in the past decade, upconverting nanoparticles are among the most promising. In this paper, we report the synthesis of upconverting nanoparticles with complex core–shell compositions, capable of being excited by 808 or 980 nm laser irradiation and exhibiting a good MRI response. The synthesized nanoparticles also demonstrated high colloidal stability in both aqueous and biological media as well as temperature-sensing capabilities, including the physiological range. Furthermore, the upconversion nanoparticles exhibited significantly lower cytotoxicity for HEK293T cells than the commercially available MRI contrast agent Gd-DTPA.

## 1 Introduction

Currently, there is an increasing demand for multifunctional nanomaterials that can be used as probes for sensitive multimodal bioimaging and targeted drug delivery. This has led to the emergence of many innovative strategies for nanotheranostics and new approaches for personalized medicine. Owing to their distinctive optical properties,^1^ such as the absence of blinking,^2^ low excitation rates, and high emission signal-to-noise ratio,^3^ rare-earth doped upconverting nanoparticles (UCNPs) have proven to be promising candidates for the detection, visualization, and treatment of various diseases.^4−9^ Moreover, UCNPs after additional doping of the UCNPs matrix with Lu^3+^, Ho^3+^, Sm^3+^, or Gd^3+^ ions could be used as multimodal contrast agents for computed tomography,^10,11^ magnetic resonance imaging,^12,13^ and single-photon emission computerized tomography.^14^ Recently, it was demonstrated that upconverting nanoparticles are also promising as remote thermal nanoprobes, particularly for monitoring treatment progress or inducing tissue inflammation, where direct temperature measurement is critical.^15^ However, despite the advanced properties of UCNPs, some important issues should be resolved before using these unique nanomaterials in biomedicine, one of which is their size. To ensure the effective clearance of UCNPs from the body, it is recommended that the particle size be reduced. Their sizes should be within the range of 6–8 nm for efficient renal elimination and 10–20 nm for hepatic clearance.^16−18^ However, reducing the size of UCNPs can affect their optical properties, that is, the quantum efficiency of the emission. Thus, it is important to find a balance among the size of nanoparticles, their optical properties, and biocompatibility. The latter issue could be solved by synthesizing nanoparticles with complex architectures, such as core–shell particles, where the outer shell could protect the optically active particle core from quenching caused by the surrounding media. Simultaneously, lattice defects are reduced, yielding a higher emission intensity. It is well established that NIR irradiation allows the excitation of particles in biological tissue transparency windows and reaches a depth of several centimeters. A considerable amount of research describing the synthesis and application of UCNPs employs 980 nm laser irradiation, but it has already been shown that at this wavelength, there is a large absorption of water in tissues; therefore, the usage of this wavelength causes unwanted thermal effects in tissues.^19^ Recently, some approaches have been developed to shift the NIR excitation to 808 nm by doping the UCNPs matrix with Yb^3+^ and Nd^3+^ as sensitizers.^19,20^ Irradiation of 808 nm falls within the first biological window where the water absorbance is ca. 90–95% lower and can penetrate deeper into biological tissues compared to the typically used wavelength of 980 nm. The appropriate selection of lanthanide ions during the synthesis of UCNPs can extend the properties of these unique particles. For instance, under excitation with an 808 nm laser, core–shell particles in which the outer shell is doped with Yb^3+^ and Nd^3+^ exhibit strong emission at 975 nm, and this is a very important feature that allows the detection of particles accumulated in biological tissues by employing noninvasive methods, such as NIR cameras. Recently, upconverting nanoparticles have been applied in nanothermometry,^21,22^ for example, for direct and local evaluation of temperature or temperature changes during therapy or in inflamed tissues.^15,23^ It has been demonstrated that Nd-doped upconverting nanoparticles can be used as nanothermometers in in vivo and in vitro systems.^24,25^ On the other hand, upconverting nanoparticles, which are additionally alloyed with gadolinium ions, provide additional magnetic properties to nanomaterials due to seven unpaired electrons in their 4*f* orbitals. Such multimodal nanoparticles could serve as contrast agents not only for optical biopsy but also for magnetic resonance imaging. Gadolinium-based contrast agents are commonly used to enhance the image contrast and detection limits in magnetic resonance imaging (MRI). However, many studies have shown that Gd-containing contrast agents are nephrotoxic, particularly in patients with renal dysfunction.^26,27^ Therefore, when constructing Gd nanoprobes, it is important to introduce the smallest possible amount of Gd into the shell of the upconverting nanoparticles. It has been shown that proton relaxation works, and better MRI contrast is ensured only if Gd ions are on the particle surface; therefore, the MRI signal and proton *T*_1_ relaxation rate strongly depend on the size, shape, and surface modification of the nanoparticles.^28−30^ Moreover, when developing new MRI contrast agents are developed, it is very important to ensure that they are biocompatible and that their toxicity is lower than that of the Gd contrast agents currently used in clinics. Several studies have shown that the toxicity of Gd-converting nanoparticles depends on their size and shape, colloidal stability, and Gd concentration.^31,32^ Moreover, it is important to note that clinically used contrast agents lack specificity and are mostly used for blood-dependent imaging, perfusion, or permeability studies. Another strategy to reduce the toxicity is the specificity of contrast agents. Nanoparticles that could precisely reach the target would have a higher local Gd ion concentration at lower total injected amount.

In this study, we present the synthesis of well-defined UCNPs with complex core–shell compositions that can be excited using both 808 and 980 nm laser irradiation. The optical and temperature-sensing properties of the UCNPs were evaluated in detail. The important properties, such as colloidal stability in aqueous and biological media as well as the viability of human kidney cells HEK 293T after exposure to UCNPs solutions of different concentrations, were investigated and compared. The availability of such promising nanomaterials as MRI contrast agents is also presented in this study.

## 2 Results and Discussion

### 2.1 Morphology and Structural Analysis

**Figure 1:**
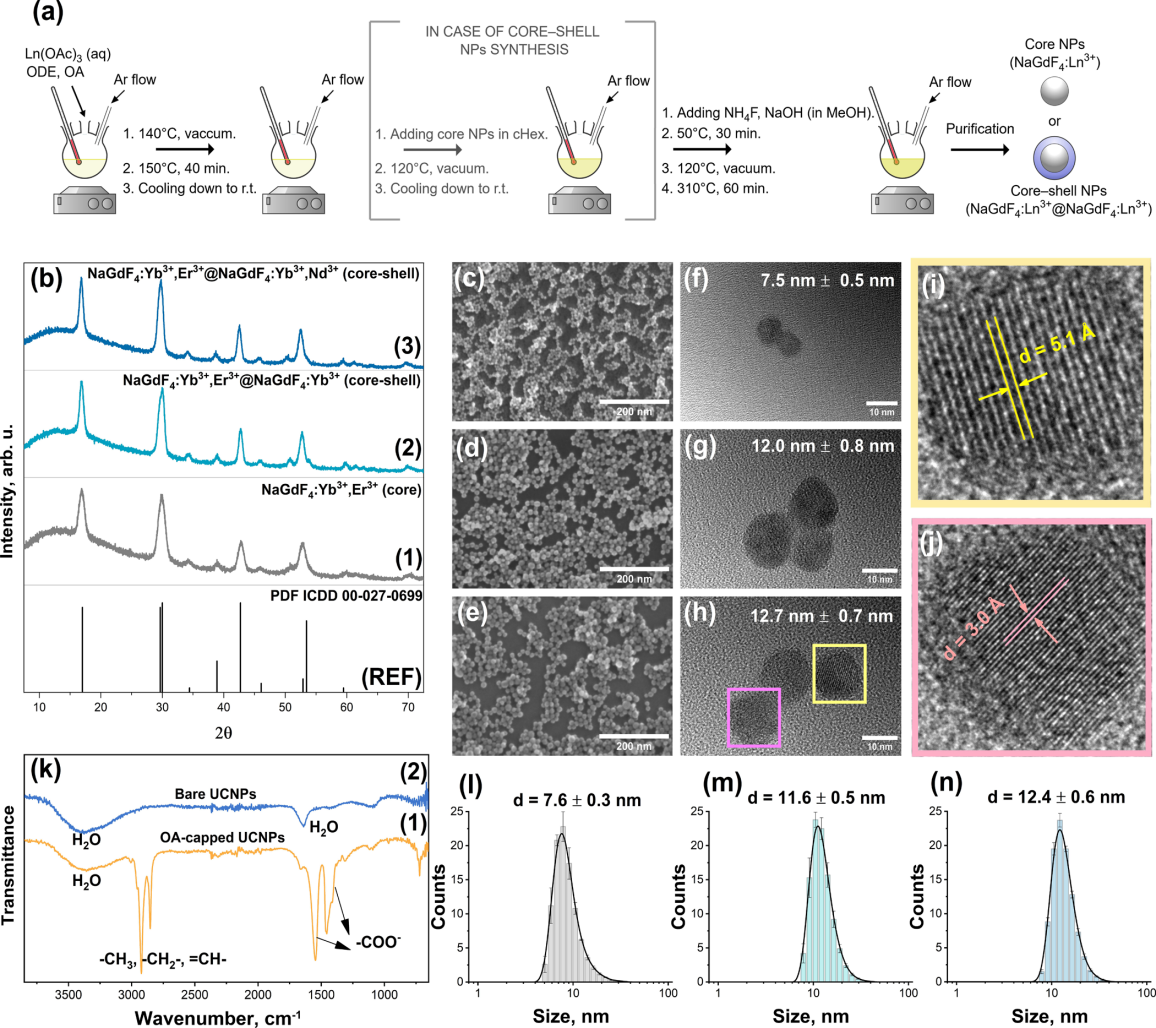
Schematic illustration of UCNPs synthesis via a coprecipitation method (a). Powder XRD patterns (b) of reference (PDF ICDD 00-027-0699), NaGdF_4_:Yb,Er (core) (1), NaGdF_4_:Yb,Er@NaGdF_4_:Yb (core–shell) (2), and NaGdF_4_:Yb,Er@NaGdF_4_:Yb,Nd (core–shell) UCNPs (3). SEM and TEM images of NaGdF_4_:Yb,Er (c,f), NaGdF_4_:Yb,Er@NaGdF_4_:Yb (d,g), and NaGdF_4_:Yb,Er@NaGdF_4_:Yb,Nd NPs (e,h). High-resolution TEM images of NaGdF_4_:Yb,Er@NaGdF_4_:Yb,Nd UCNPs (i,j) showing different interplanar distances. FT-IR spectra of NaGdF_4_:Yb,Er@NaGdF_4_:Yb,Nd UCNPs before and after ligand removal (k). Particle size distribution (PSD) (from DLS measurements) of NaGdF_4_:Yb,Er (l), NaGdF_4_:Yb,Er@NaGdF_4_:Yb (m), and NaGdF_4_:Yb,Er@NaGdF_4_:Yb,Nd UCNPs (n) suspended in DI water.

The core NaGdF_4_:18%Yb^3+^,2%Er^3+^ (NaGdF_4_:Yb,Er), core–shell particles NaGdF_4_:18%Yb^3+^,2%Er^3+^@NaGdF_4_:5%Yb^3+^ (NaGdF_4_:Yb,Er@NaGdF_4_:Yb), and NaGdF_4_:18%Yb^3+^,2%Er^3+^@NaGdF_4_:5%Yb^3+^,40%Nd^3+^ (NaGdF_4_:Yb,Er@NaGdF_4_:Yb,Nd) were synthesized via a coprecipitation synthesis route according to the previously published data with minor adjustments^33^ (for the detailed synthesis and analysis procedures, please refer to SI). The schematic illustration of the core and core–shell UCNPs synthesis is provided in Figure 1a. The powder X-ray diffraction (XRD) analysis of the synthesized UCNPs indicated that samples are of the β-NaGdF_4_ hexagonal crystal structure (space group *P*6_3_/*mmc*) (PDF ICDD 00-027-0699). No additional peaks from the impurity phases were detected in the recorded patterns (see Figure 1b). The particle size and particle size distribution (PSD) were evaluated from SEM (please refer to Figure 1c–e) and TEM (please refer to Figure 1f–h) images using ImageJ software (v.1.53m) (135 random particles were selected for calculations). The synthesized particles are unimodal with the calculated size of 7.5 ± 0.5 nm for NaGdF_4_:Yb,Er core particles, 12.0 ± 0.8 nm for NaGdF_4_:Yb,Er@NaGdF_4_:Yb, and 12.7 ± 0.7 nm for NaGdF_4_:Yb,Er@NaGdF_4_:Yb,Nd core–shell particles.

Figure 1i,j shows the interplanar distances of the NaGdF_4_:Yb,Er@NaGdF_4_:Yb,Nd core–shell nanoparticles. The calculated lattice spacings between (100) and (101) crystal planes are 5.1 and 3.0 Å, respectively, and correspond well with the particle’s XRD pattern. Figure 1k depicts the FT-IR spectra of NaGdF_4_:Yb,Er@NaGdF_4_:Yb,Nd UCNPs before and after the oleate ligand removal. It is important to note that the UCNPs after synthesis are oleic-capped and are not dispersible in water. An additional ligand removal procedure is necessary to disperse the UCNPs in aqueous media. For a detailed procedure of oleic acid ligand removal from the UCNPs surface, please refer to the SI. The recorded FT-IR spectra show that the OA-capped UCNPs have typical absorption peaks in the ranges of 1400–1600 and 2800–3000 cm^–1^, which can be attributed to −COO^–^ and C–H vibrations, respectively. After ligand removal, no traces of organic compounds were detected in the FT-IR spectra of the UCNPs. The broad absorption bands peaks at ca. 1650 and 3450 cm^–1^ were assigned to the vibrations of water molecules.

After the removal of the OA ligands, the UCNPs size was also evaluated by DLS measurements (see Figure 1l–n). The obtained particle size for NaGdF_4_:Yb,Er, NaGdF_4_:Yb,Er@NaGdF_4_:Yb, and NaGdF_4_:Yb,Er@NaGdF_4_:Yb,Nd UCNPs was 7.6 ± 0.3, 11.6 ± 0.5, and 12.4 ± 0.6 nm, respectively. These values match exceptionally well with those calculated from TEM images.

### 2.2 Optical Properties

**Figure 2:**
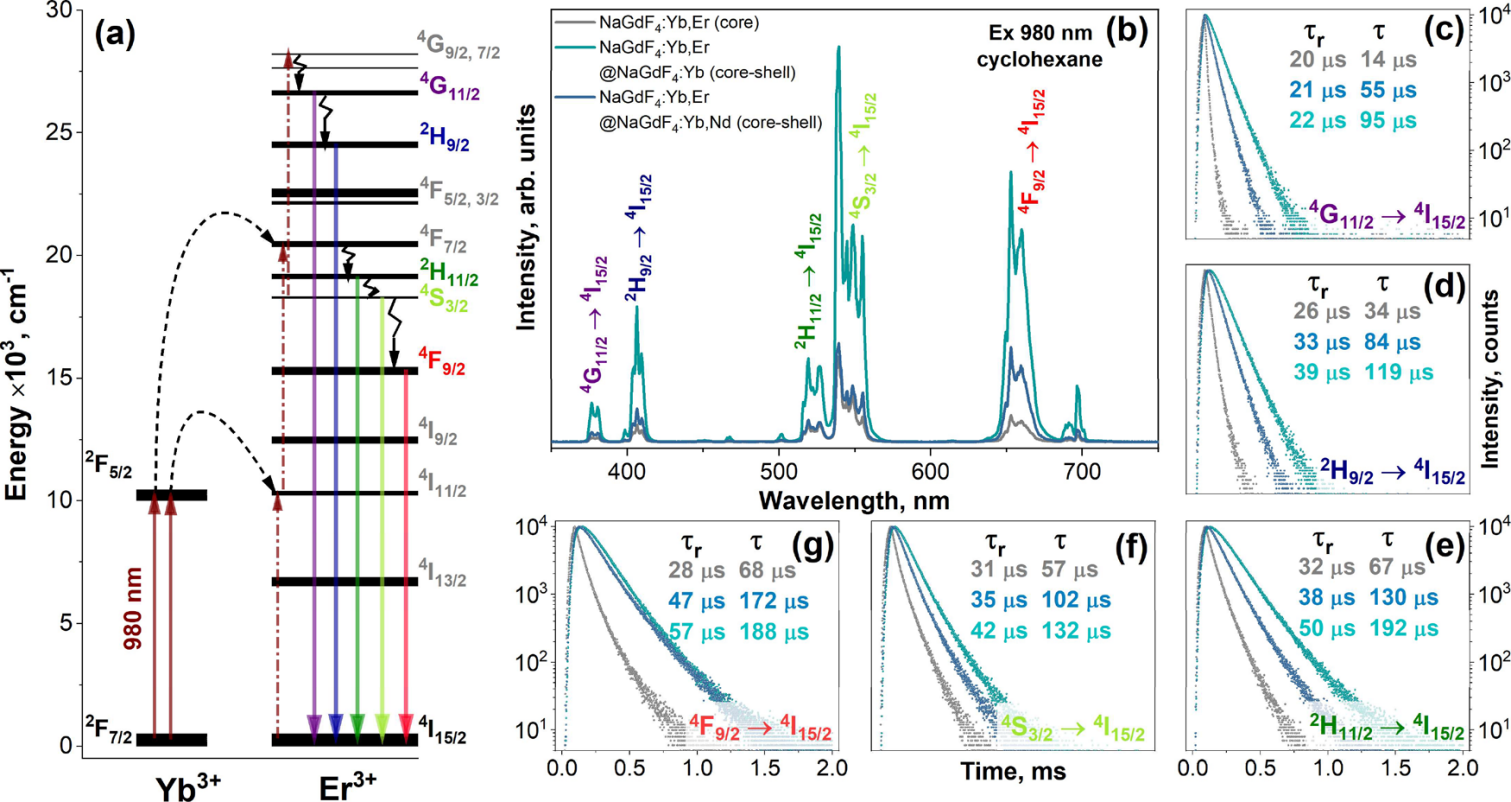
Simplified energy-level diagram of Yb^3+^ and Er^3+^ (a); emission spectra of UCNPs (core or core–shell with different compositions) dispersed in cyclohexane (b); PL decay curves with calculated UC emission rise time (τ_r_) and UC lifetime (τ) values for different emission transitions: ^4^G_11/2_ → ^4^I_15/2_ (c), ^2^H_9/2_ → ^4^I_15/2_ (d), ^2^H_11/2_ → ^4^I_15/2_ (e), ^4^S_3/2_ → ^4^I_15/2_ (f), and ^4^F_9/2_ → ^4^I_15/2_ (g).

A simplified diagram of the Yb^3+^ and Er^3+^ energy levels involved in the upconversion process is shown in Figure 2a. Yb^3+^ possesses a much higher absorption cross-section for 980 nm radiation compared to Er^3+^.^34^ Therefore, Yb^3+^ absorbs the 980 nm laser radiation (^2^F_7/2_ → ^2^F_5/2_ transition) and transfers the energy to Er^3+^, which emits in the near-UV to vis spectral range after receiving two or more consecutive energy transfers. The UC emission spectra of the OA-capped core and core–shell UCNPs were recorded in cyclohexane (please refer to Figure 2b), whereas those of the bare (with removed OA ligands) UCNPs were measured in DI water (please refer to Figure S1b). Here, all samples were excited with a continuous wave 980 nm laser. In both cases, the measurement conditions (emission bandwidth, step size, integration time, etc.) were kept as close as possible. The UC emission spectra of the measured samples contained several sets of typical Er^3+^ emission lines attributed to the ^4^G_11/2_ → ^4^I_15/2_ (ca. 377 nm), ^2^H_9/2_ → ^4^I_15/2_ (ca. 407 nm), ^2^H_11/2_ → ^4^I_15/2_ (ca. 519 nm), ^4^S_3/2_ → ^4^I_15/2_ (ca. 539 nm), and ^4^F_9/2_ → ^4^I_15/2_ (ca. 653 nm) transitions. As expected, the emission of NaGdF_4_:Yb,Er@NaGdF_4_:Yb and NaGdF_4_:Yb,Er@NaGdF_4_:Yb,Nd core–shell UCNPs is more intensive than in NaGdF_4_:Yb,Er core nanoparticles. The formed outer shell reduces surface defects and protects the optically active core from quenching induced by the surrounding media, resulting in a higher upconversion intensity.^35^ The evolution of the sum upconversion emission intensity of the core and core–shell UCNPs in different media is summarized in Table 1.

**Table 1:** Evolution of the UC Sum Emission Intensity of UCNPs in Cyclohexane and DI Water (λ_ex_ = 980 nm Laser)

	cyclohexane	DI water
NaGdF_4_:Yb,Er core	*I*_em_^a^	*I*_em_/12.3
NaGdF_4_:Yb,Er@NaGdF_4_:Yb	6.7 × *I*_em_	(6.7 × *I*_em_)/2
NaGdF_4_:Yb,Er@NaGdF_4_:Yb,Nd	1.9 × *I*_em_	(1.9 × *I*_em_)/2

a *I*_em_ is the sum emission intensity of the NaGdF_4_:Yb,Er core NPs in cyclohexane.

For easier comparison of the sum emission intensities (area under the emission spectra), the sum emission intensity of NaGdF_4_:Yb,Er core UCNPs in cyclohexane was selected as a reference and marked as *I*_em_. After ligand removal and redispersion of UCNPs in DI water, the upconversion emission of the core NaGdF_4_:Yb,Er UCNPs was barely detectable (please refer to Figure S1b). In this case, emission quenching is induced by water molecules, and the sum emission intensity of the core NaGdF_4_:Yb,Er nanoparticles dispersed in DI water is about 12.3 times lower than that dispersed in cyclohexane. It turned out that the chemical composition of the outer shell also has a significant effect on the core–shell UCNPs emission intensity. For instance, the NaGdF_4_:Yb shell increased the emission intensity by ca. 6.7 times, whereas the NaGdF_4_:Yb,Nd shell increased the emission intensity by only ca. 1.9 times compared with the core UCNPs dispersed in cyclohexane. After redispersing NaGdF_4_:Yb,Er@NaGdF_4_:Yb and NaGdF_4_:Yb,Er@NaGdF_4_:Yb,Nd core–shell UCNPs in DI water, the sum emission intensity decreased twice in both cases (please refer to Table 1). It is important to note that the sum emission intensity of NaGdF_4_:Yb,Er@NaGdF_4_:Yb,Nd NPs is ca. 3.5 lower compared to that of the NaGdF_4_:Yb,Er@NaGdF_4_:Yb NPs ((6.7 × *I*_em_)/(1.9 × *I*_em_)). The reduced emission intensity of the UCNPs containing Nd^3+^ in the outer shell is probably caused by the absorption of the Er^3+^ emission by Nd^3+^. Neodymium ions have a complex energy-level structure and possess many absorption bands over the entire visible spectrum.^36^ Moreover, we also observed that the red emission band (^4^F_9/2_ → ^4^I_15/2_) of Er^3+^ is less affected by the presence of Nd^3+^ than the emission bands in the green and violet spectral areas because Nd^3+^ virtually does not absorb at ca. 650 nm. This explanation is supported by the measured PL decay curves and calculated PL lifetimes of different Er^3+^ emission transitions (please refer to Figure 2c–g). The PL decay curves became steeper for the ^4^G_11/2_ → ^4^I_15/2_ (c), ^2^H_9/2_ → ^4^I_15/2_ (d), ^2^H_11/2_ → ^4^I_15/2_ (e), and ^4^S_3/2_ → ^4^I_15/2_ (f) transitions if Nd^3+^ was present in the outer shell, indicating a decrease in the PL lifetime values. However, this was not true for the ^4^F_9/2_ → ^4^I_15/2_ (g) transition. Here, the PL decay curves of the NaGdF_4_:Yb,Er@NaGdF_4_:Yb and NaGdF_4_:Yb,Er@NaGdF_4_:Yb,Nd were almost identical, and the calculated PL lifetime values were very similar.

**Figure 3:**
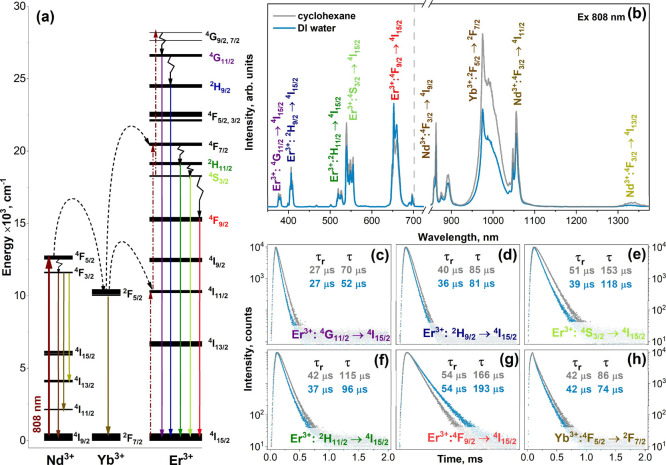
Energy-level scheme and optical transitions of Nd^3+^, Yb^3+^, and Er^3+^ (a); emission spectra of NaGdF_4_:Yb,Er@NaGdF_4_:Yb,Nd core–shell UCNPs in the visible and NIR regions (b); PL decay curves with calculated UC emission rise time and UC emission lifetime values for different emission transitions: ^4^G_11/2_ → ^4^I_15/2_ (c), ^2^H_9/2_ → ^4^I_15/2_ (d), ^2^H_11/2_ → ^4^I_15/2_ (e), ^4^S_3/2_ → ^4^I_15/2_ (f), ^4^F_9/2_ → ^4^I_15/2_ (g), and ^2^F_5/2_ → ^2^F_7/2_ (Yb^3+^) (h) of UCNPs dispersed in DI water or cyclohexane under 808 nm laser excitation.

Usually, upconverting nanoparticles are excited with 980 nm laser radiation.^37^ However, such radiation is absorbed by water in biological tissues and causes undesirable heating, which, of course, is a significant drawback in practical applications. Excessive heating can cause the denaturation of proteins within cells and other side effects. On the other hand, 808 nm NIR radiation falls within the first biological window and penetrates deeper into biological tissues than 980 nm radiation.^38^ Therefore, UCNPs that can be excited with 808 nm radiation are of great interest. Yb^3+^ itself does not absorb 808 nm radiation, which was confirmed by measuring the UC emission spectra of the NaGdF_4_:Yb,Er core and NaGdF_4_:Yb,Er@NaGdF_4_:Yb UCNPs under 808 nm laser excitation (please refer to Figure S2). The intensity of the given emission spectra was very weak and resulted only from the direct excitation of the Er^3+^ energy levels. However, in combination with Nd^3+^, a highly efficient energy-transfer system was obtained. Nd^3+^ possesses up to 1 order of magnitude higher absorption cross-section (ca. 10^–19^ cm^2^) for 808 nm radiation than the Yb^3+^ absorption cross-section for 980 nm radiation.^39^ In this case, Nd^3+^ efficiently absorbs 808 nm radiation, transfers it to Yb^3+^, which subsequently transfers it to Er^3+^. A simplified scheme of the Nd^3+^, Yb^3+^, and Er^3+^ energy levels involved in the discussed energy-transfer process is shown in Figure 3a.

The emission spectra (808 nm laser excitation) of NaGdF_4_:Yb,Er@NaGdF_4_:Yb,Nd UCNPs dispersed in cyclohexane and DI water are shown in Figure 3b. Typical emission bands of Er^3+^ similar to those presented in Figure 2b were observed. The given spectra also show that in addition to the typical Er^3+^ emission bands in the UV–vis region, the emission bands of Yb^3+^ and Nd^3+^ in the NIR region were also observed. The emission bands at ca. 865, 1055, and 1335 nm are attributed to the ^4^F_3/2_ → ^4^I_9/2_, ^4^F_3/2_ → ^4^I_11/2_, and ^4^F_3/2_ → ^4^I_13/2_ transitions of Nd^3+^, respectively, whereas the intense emission at 975 nm is attributed to the ^2^F_5/2_ → ^2^F_7/2_ transition of Yb^3+^.

The dependence of the NaGdF_4_:Yb,Er@NaGdF_4_:Yb,Nd UCNPs emission on the 808 nm laser power is shown in Figure S3a. It turned out that the UC emission intensity gradually decreased with decreasing laser power from 2 to 0.4 W. These data were also used to determine the number of 808 nm wavelength photons involved in the UC emission of Er^3+^ in different UV–vis regions. The logarithmic integrated emission of a certain transition was plotted as a function of the logarithmic laser power, and the number of photons was determined from the slope of the linear approximation (see Figure S3b). The obtained slope values for the ^4^G_11/2_ → ^4^I_15/2_, ^2^H_9/2_ → ^4^I_15/2_, ^2^H_11/2_ + ^4^S_3/2_ → ^4^I_15/2_, and ^4^F_9/2_ → ^4^I_15/2_ transitions are 3.13, 2.79, 2.33, and 2.10, respectively. Therefore, it can be concluded that three 808 nm wavelength photons are involved in the ^4^G_11/2_ → ^4^I_15/2_ and ^2^H_9/2_ → ^4^I_15/2_ transitions, whereas only two are required for the ^2^H_11/2_ + ^4^S_3/2_ → ^4^I_15/2_ and ^4^F_9/2_ → ^4^I_15/2_ emission transitions. It was also observed that for all investigated emission transitions at higher laser powers (>1 W), the emission intensity deviates from the linear trend, bends, and finally reaches a plateau at a laser power ca. 2 W. This shows that at these laser powers, the saturation point was reached.

Notably, the emission bands of Nd^3+^ and Yb^3+^ in the NIR region could also be useful in optical imaging because most of them fall within the second biological window (from 1000 to 1700 nm).^40^ Therefore, such UCNPs could even be detected in vivo using NIR cameras.^41,42^ It was also observed that the upconversion emission intensity of the NaGdF_4_:Yb,Er@NaGdF_4_:Yb,Nd UCNPs in the UV–vis range was almost the same, regardless of whether the UCNPs were dispersed in cyclohexane or DI water (please refer to Figure 3b). However, it was also observed that the emission intensity ratio between the transitions in the red and green spectral areas was not the same if the UCNPs were dispersed in cyclohexane or DI water. For instance, the calculated green-to-red (G/R) ratios for NaGdF_4_:Yb,Er@NaGdF_4_:Yb,Nd UCNPs, dispersed in cyclohexane and DI water, are 1.05 and 0.65, respectively. This indicates that the population of the ^4^F_9/2_ level of Er^3+^ increased when UCNPs were dispersed in water. The mechanism of this phenomenon was explained by Resch-Genger and co-workers.^43^ The changes in the emission spectra were also reflected in the PL decay curves (please refer to Figure 3c–h). The calculated PL lifetime values of most energy transitions (under excitation with an 808 nm laser) for NaGdF_4_:Yb,Er@NaGdF_4_:Yb,Nd core–shell UCNPs dispersed in cyclohexane were higher than those of UCNPs dispersed in DI water. However, this is not the case with the ^4^F_9/2_ → ^4^I_15/2_ transition of Er^3+^ at about 653 nm, where the PL lifetime is longer if UCNPs are dispersed in water.

### 2.3 Particle Colloidal Stability Measurements

**Figure 4:**
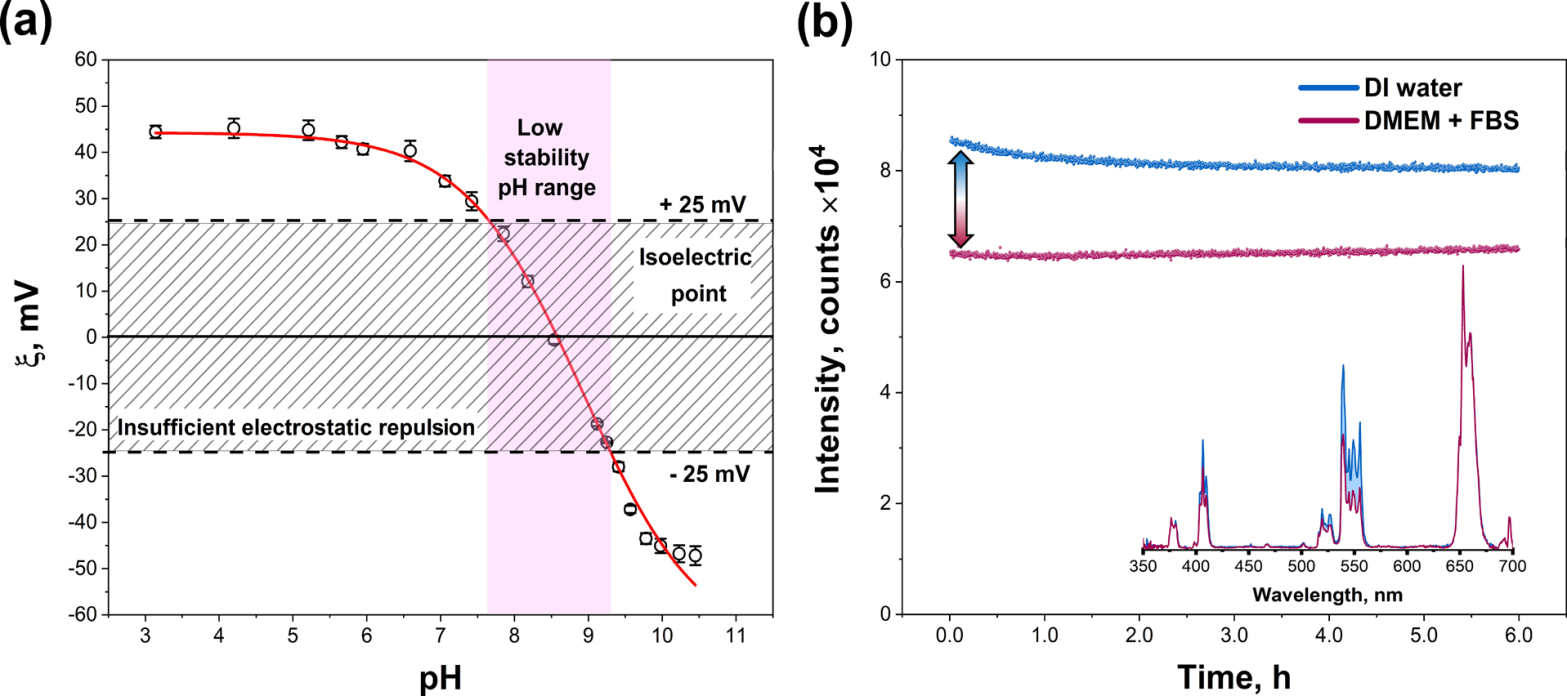
Zeta potential of ligand-free NaGdF_4_:Yb,Er@NaGdF_4_:Yb,Nd UCNPs in water as a function of pH (a) and colloidal stability of NaGdF_4_:Yb,Er@NaGdF_4_:Yb,Nd UCNPs in DI water and DMEM media supplemented with 10% FBS evaluated as change of emission (λ_em_ = 539.5 nm) intensity in time (b).

It is well established that if the absolute of zeta potential (ζ) determined for colloid systems exceeds ±25 mV, the electrostatic repulsion forces overdominate the forces caused by van der Waals and could ensure efficient colloidal stability.^44,45^ To evaluate the colloidal stability of ligand-free UCNPs in water, the zeta potential as a function of pH was measured, and the obtained results are presented in Figure 4a. We want to emphasize that the zeta potentials of all UCNPs were similar, regardless of their chemical composition. Thus, the most promising NaGdF_4_:Yb,Er@NaGdF_4_:Yb,Nd core–shell UCNPs were selected for further investigation. It was determined that UCNPs possess an isoelectric point (IEP) in the slightly basic region (pH = 8.4). Figure 4a also demonstrates that the NaGdF_4_:Yb,Er@NaGdF_4_:Yb,Nd UCNPs are not stable in the 7.6 < pH < 9.3 range, since |ζ| < 25 mV, and are perfectly stable at pH < 7.6 or pH > 9.3, since |ζ| > 25 mV.

Colloidal stability measurements of NaGdF_4_:Yb,Er@NaGdF_4_:Yb,Nd UCNPs in deionized (DI) water and Dulbecco’s modified Eagle's medium (DMEM) supplemented with 10% of fetal bovine serum (FBS) (please refer to Figure 4b) were initially performed for 6 h, followed by 24 h experiment at a constant 25 °C temperature. Particle stability was evaluated from the intensity change of the ^4^S_3/2_ → ^4^I_15/2_ (λ_max_ = 539.5 nm) emission transition over time. Figure 4b demonstrates that the upconversion intensity of bare NaGdF_4_:Yb,Er@NaGdF_4_:Yb,Nd core–shell UCNPs dispersed in DI water dropped down by only 6% of its initial value within 6 h and then remained unchanged for a period of 24 h (please refer to Figure S4a in SI). In contrast, the intensity of the bare NaGdF_4_:Yb,Er@NaGdF_4_:Yb,Nd UCNPs in DMEM supplemented with 10% FBS showed perfect stability over a period of 24 h as no drop in the initial emission intensity was observed (please refer to Figures 4b and S4b in SI). However, we also want to point out that the initial intensity of UCNPs emission in DMEM + FBS media was lower than that measured in DI water. The reduced initial intensity is caused by the absorption of media and the higher scattering of serum proteins, which is typically observed in the range of 600 nm. Furthermore, the initial UC emission intensities of NaGdF_4_:Yb,Er@NaGdF_4_:Yb,Nd UCNPs dispersed in DI water and DMEM + FBS were the same for lower energy transitions (^4^F_9/2_ → ^4^I_15/2_, λ_em_ = 653 nm) (please refer to the inset in Figure 4b). To conclude, we emphasize that such particle stability in aqueous and biological media is sufficient for in vitro measurement in cells.

### 2.4 Optical Nanothermometry

**Figure 5:**
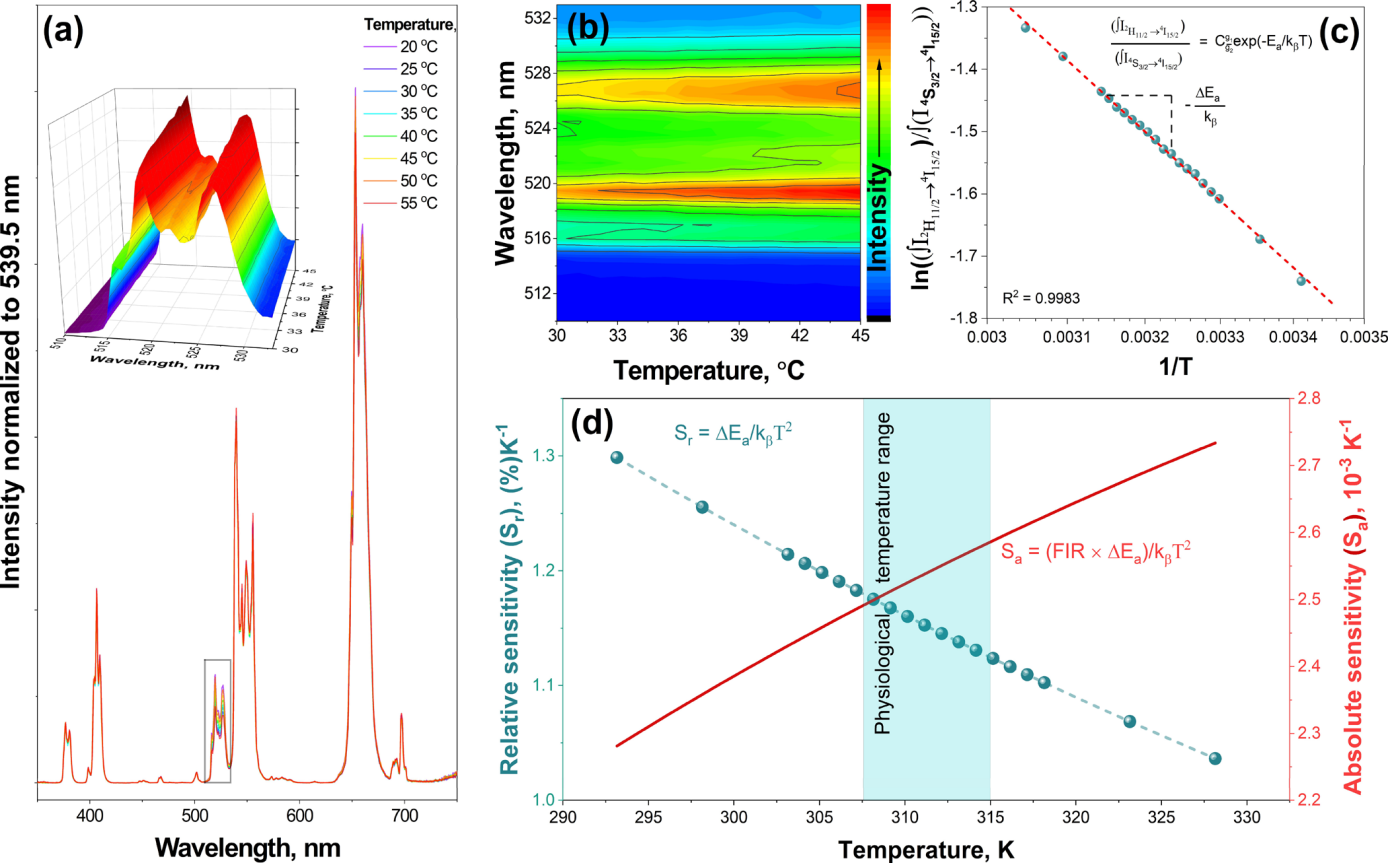
Temperature-dependent emission spectra (λ_ex_ = 808 nm laser) of NaGdF_4_:Yb,Er@NaGdF_4_:Yb,Nd (a) and emission intensity contour plot (range 510–545 nm) as a function of temperature (b). Logarithmic ratio of sum intensity of ^2^H_11/2_ → ^4^I_15/2_ and ^4^S_3/2_ → ^4^I_15/2_ emission transitions as a function of 1/*T* (c); relative (*S*_r_, blue dots) and absolute (*S*_a_, red line) sensitivity as a function of temperature (d).

The Er^3+^ emission in the green spectral region is unique because it contains two thermally coupled energy levels, ^2^H_11/2_ and ^4^S_3/2_.^46^ The energy difference between these levels is approximately 700–800 cm^–1^.^47^ Therefore, Er^3+^-doped materials can be used as temperature sensors (Boltzmann thermometers).^48^ In the literature, the sensing ability of Er^3+^-doped solid-state materials is typically measured over a wide temperature range (77–500 K).^49^ It was also shown that at temperatures lower than 150 K, the emission from the spin-forbidden ^2^H_11/2_ → ^4^I_15/2_ transition was not detectable but was observed as the temperature increased because of the energy transfer from the thermally coupled ^4^S_3/2_ level. As demonstrated in the previous section, our prepared NaGdF_4_:Yb,Er@NaGdF_4_:Yb,Nd UCNPs showed sufficient colloidal stability; therefore, the thermosensing properties of these UCNPs in aqueous solutions under 808 nm laser excitation were investigated. Figure 5a shows the temperature-dependent emission spectra (normalized to 539.5 nm) of the NaGdF_4_:Yb,Er@NaGdF_4_:Yb,Nd core–shell UCNPs. It is obvious that the change in temperature, even in such a relatively narrow temperature range (including physiological temperature), affects the fluorescence intensity ratio between spin-forbidden ^2^H_11/2_ → ^4^I_15/2_ and spin-allowed ^4^S_3/2_ → ^4^I_15/2_. It should also be noted that the intensities of the other Er^3+^ emission transitions were virtually the same in the 20–25 °C temperature range. The increase in the ^2^H_11/2_ → ^4^I_15/2_ intensity as a function of temperature is plotted as a 3D surface color map (see the inset in Figure 5a) and contour graph (please refer to Figure 5b). The integrated emission intensities of the ^2^H_11/2_ → ^4^I_15/2_ and ^4^S_3/2_ → ^4^I_15/2_ transitions correspond to the populations of these levels following the Boltzmann distribution (eq 1):^50^
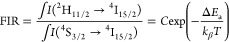
1where FIR is the fluorescence intensity ratio between the ^2^H_11/2_ → ^4^I_15/2_ and ^4^S_3/2_ → ^4^I_15/2_ transitions, *C* is a constant, Δ*E*_a_ is the effective energy difference between the two thermally coupled energy levels, *k*_β_ is the Boltzmann constant (8.617342 × 10^–5^ eV/K or 0.695034800 cm^–1^/K),^51,52^ and *T* is the absolute temperature. The logarithmic ratios of the ^2^H_11/2_ → ^4^I_15/2_ and ^4^S_3/2_ → ^4^I_15/2_ integrated intensities as a function of the reverse absolute temperature (1/*T*) are plotted in Figure 5c. A linear trend was observed, and the effective energy difference (Δ*E*_a_) between the ^2^H_11/2_ and ^4^S_3/2_ thermally coupled levels was calculated from the slope and was equal to 775 ± 5 cm^–1^. In order to apply UCNP’s to sensing, the relative (*S*_r_) and absolute (*S*_a_) sensitivities must be evaluated.^53^
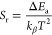
2
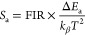
3*S*_r_ represents the rate at which the FIR changes with a certain change in the temperature. This also indicates that the FIR sensitivity increases when the energy gap between two thermally coupled levels increases.^50^*S*_r_ values are used to compare the temperature-sensing abilities of materials with different chemical compositions, crystal structures, and synthesized using various techniques.^54^ The *S*_a_ values, in turn, show the resolution of the measured temperature. The *S*_r_ and *S*_a_ values obtained for the NaGdF_4_:Yb,Er@NaGdF_4_:Yb,Nd core–shell UCNPs are plotted in Figure 5d. The calculated *S*_r_ and *S*_a_ values for NaGdF_4_:Yb,Er@NaGdF_4_:Yb,Nd core–shell UCNPs at 37 °C (310.15 K) (in the physiological temperature range) were 1.16% K^–1^ and 2.52 × 10^–3^ K^–1^, respectively. Furthermore, at 303 K, *S*_r_ is 1.20% K^–1^, which is in good agreement with reported literature results, where the calculated *S*_*r*_ is similar and varies in the range of 1.11–1.24% K^–1^ at the same temperature.^47,55,56^ The same experiment was conducted by using 980 nm laser irradiation. A comparison of the thermosensing properties of UCNPs under excitation by 980 and 808 nm laser irradiation is presented in the ESI as Figure S5. The normalized fluorescence intensity ratio (FIR) between the integrated areas of the ^2^H_11/2_ → ^4^I_15/2_ and ^4^S_3/2_ → ^4^I_15/2_ energy transitions in Er^3+^ under excitation with 808 and 980 nm lasers was virtually identical (Figure S5e). The calculated relative sensitivities (*S*_r_) at 298.15 K (25 °C) for UCNPs excited using 808 and 980 nm lasers were 1.28% K^–1^ and 1.36% K^–1^, respectively. This clearly indicates that NaGdF_4_:Yb,Er@NaGdF_4_:Yb,Nd UCNPs can serve as effective temperature sensors for bioapplications.

### 2.5 Cell Viability and MRI Measurements

**Figure 6:**
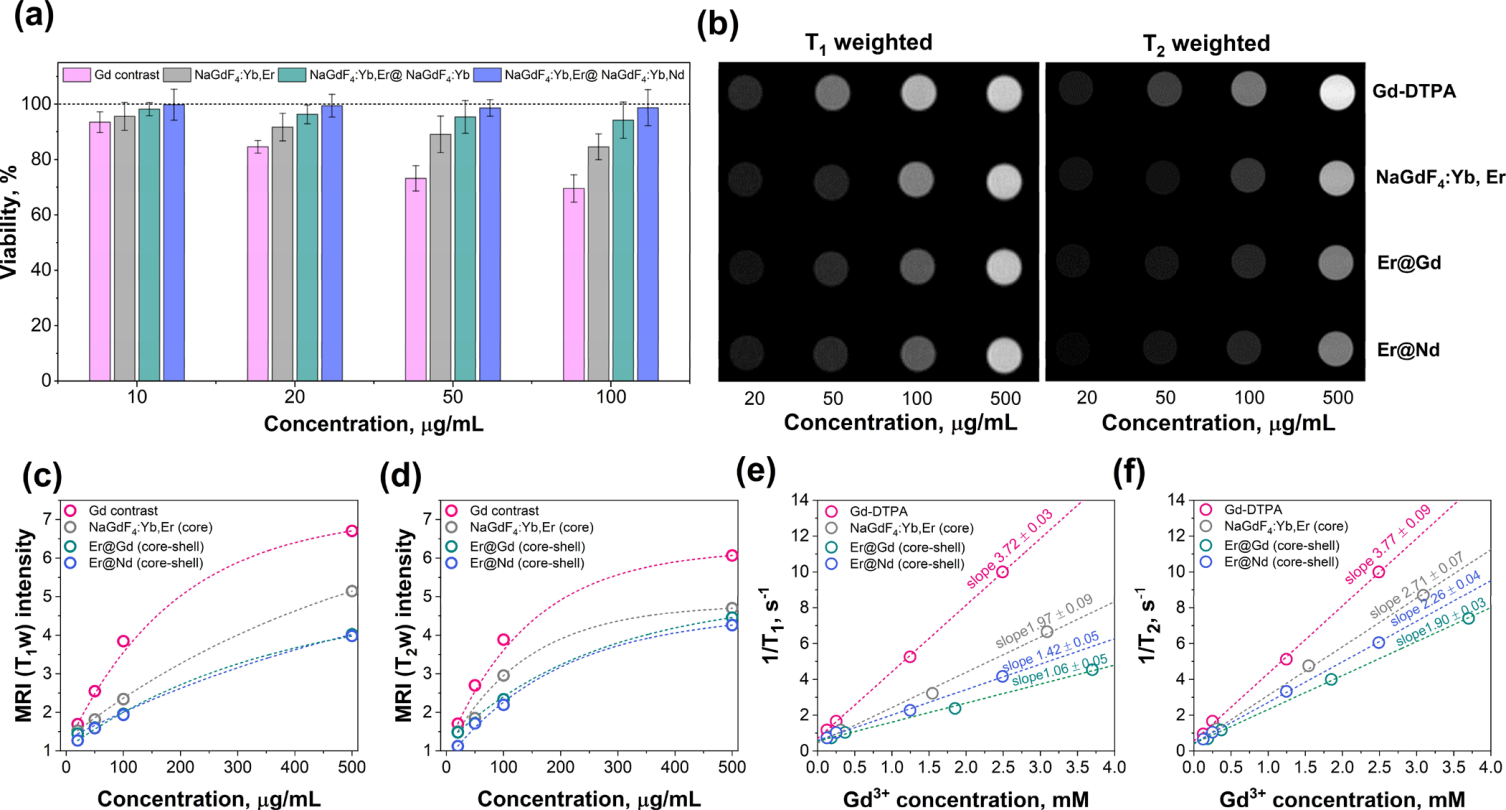
Viability of HEK 293T kidney cells exposed to different concentrations of UCNPs and the commercial MRI contrast agent Gd-DTPA (a); MRI signal intensity greyscale comparison of commercial gadolinium complex (Gd-DTPA) and UCNPs with different architectures and compositions at various concentrations (b); longitudinal *T*_1_w (c) and transverse *T*_2_w (d) MRI intensity as a function of NPs concentration (μg/mL); longitudinal (1/*T*_1_) (e) and transverse (1/*T*_2_) (f) relaxivity plotted as a function of molar Gd^3+^ concentration. The slopes in parts (e) and (f) represent the molar relaxivities of the UCNPs and Gd-DTPA, respectively. Please note that the black color in the grayscale images represents the MRI response of pure DI water at 37 °C.

Human embryonic kidney cells HEK 293T were used to evaluate the cytotoxicity of the synthesized UCNPs. The viability of kidney cells exposed to different concentrations (10–100 μg/mL) of UCNPs and the commercial MRI contrast agent Gd-DTPA was tested. After 24 h of exposure to UCNPs solutions, the viability of the human kidney cells HEK 293t was evaluated using the XTT method (for a detailed description of the XTT method, please refer to SI). The viability results of triplicate experiments are presented in Figure 6a. It was observed that UCNPs, regardless of their chemical composition or architecture, showed very low toxicity compared to the commercial Gd-complex at the same concentrations. The viability of human kidney cells HEK 293t after exposure to the 100 μg/mL concentration of NaGdF_4_:Yb,Er, NaGdF_4_:Yb,Er@NaGdF_4_:Yb, and NaGdF_4_:Yb,Er@NaGdF_4_:Yb,Nd UCNPs remains 85, 94, and 99%, respectively. Meanwhile, the cell viability decreased to 69% when Gd-DTPA was used. The obtained results could be related to the solubility, size, and composition of the UCNPs. The dissolution of UCNPs in aqueous solutions is relatively low compared to that of Gd-chelates;^57^ thus, in our opinion, the size and composition of UCNPs are the most important factors causing cell toxicity. The core NaGdF_4_:Yb,Er are the smallest UCNPs used in this study; therefore, they possess the highest surface area and the highest amount of Gd^3+^ exposed to the surface, leading to the possible release of Gd^3+^ ions and damage to the cells. In turn, the size of NaGdF_4_:Yb,Er@NaGdF_4_:Yb and NaGdF_4_:Yb,Er@NaGdF_4_:Yb,Nd core–shell particles is approximately the same; hence, the toxicity of UCNPs in such particular cases mostly depends on the NPs chemical composition. The outer shell of NaGdF_4_:Yb,Er@NaGdF_4_:Yb contains 95 mol % of Gd^3+^, whereas the outer shell of NaGdF_4_:Yb,Er@NaGdF_4_:Yb,Nd contains only 55 mol % Gd^3+^. At the same time, the latter UCNPs are less toxic than the former (please refer to Figure 6a), leading to the conclusion that the toxicity caused by Gd^3+^ is higher than that of other lanthanide ions (Yb^3+^, Nd^3+^) in the composition of UCNPs.

The suitability of UCNPs for MRI applications was assessed by taking *T*_1_-weighted and *T*_2_-weighted images of UCNPs solutions with concentrations ranging from 20 to 500 μg/mL at human body temperature (37 °C). The results were compared to those of the clinically used reference compound gadopentetic acid (Gd-DTPA). *T*_1_-weighted and *T*_2_-weighted MR coronal slice images are shown in Figure 6b. The MR signal intensity values (grayscales) as a function of different particle concentrations are shown in Figure 6c,d. Positive MRI signals (*T*_1_ and *T*_2_ weighted) were visible at all applied concentration ranges for all contrast materials used. The *T*_1_-weighted MRI signal intensity of Gd-DTPA, NaGdF_4_:Yb,Er, NaGdF_4_:Yb,Er@NaGdF_4_:Yb, and NaGdF_4_:Yb,Er@NaGdF_4_:Yb,Nd contrast materials at the highest applied concentration (500 μg/mL) is 6.7, 5.1, 4.0, and 4.0 times higher compared to the MRI signal of DI water (please refer to Figure 6c). Similar results were also observed for the *T*_2_-weighted MRI signal; in such case, the MRI signal of Gd-DTPA, NaGdF_4_:Yb,Er, NaGdF_4_:Yb,Er@NaGdF_4_:Yb, and NaGdF_4_:Yb,Er@NaGdF_4_:Yb,Nd contrast materials is 6.1, 4.7, 4.5, and 4.3 times higher than the MRI signal of DI water (please refer to Figure 6d). The similar MRI signal intensity of UCNPs compared to the reference contrast agent Gd-DTPA and the low toxicity revealed that such promising functional materials could be applied for further MRI investigations in vivo.

For a more detailed characterization, the longitudinal (*T*_1_) and transversal (*T*_2_) relaxivity at a 1.5T magnetic field was measured by applying inversion recovery and turbo spin echo methods. The *T*_1_ and *T*_2_ proton relaxation times (1/*T*_1_ and 1/*T*_2_) were measured at 37 °C as a function of the MRI agent concentration. In these cases, the concentration of the particles was expressed as the Gd ion concentration, calculated according to the UCNPs composition. The 1/*T*_1_ and 1/*T*_2_ values as a function of Gd^3+^ molar concentration for all samples used in this study were plotted and approximated linearly (please refer to Figure 6e,f). The slope of the line indicates the molar relaxivity (*r*). The calculated longitudinal (*r*_1_) and transverse (*r*_2_) molar relaxivity values (s^–1^ per mM Gd^3+^) are 3.72 and 3.77 for Gd-DTPA; 1.97 and 2.71 for NaGdF_4_:Yb,Er; 1.06 and 1.90 for NaGdF_4_:Yb,Er@NaGdF_4_:Yb; and 1.42 and 2.26 for NaGdF_4_:Yb,Er@NaGdF_4_:Yb,Nd.

Gd-DTPA exhibited the highest *r*_1_ and *r*_2_ values for all of the contrast materials used. This means that Gd-DTPA has the strongest interaction with water molecules and exhibits the shortest relaxation times, giving the highest MRI signal intensity using the selected registration protocol parameters (for more details, please refer to SI). The MRI response of UCNPs, in turn, is dependent on the particle composition and architecture. The smallest core NaGdF_4_:Yb,Er UCNPs (ca. 7 nm) used in this study possessed stronger MRI response compared to core–shell counterparts (NaGdF_4_:Yb,Er@NaGdF_4_:Yb or NaGdF_4_:Yb,Er@NaGdF_4_:Yb,Nd). Multiple factors can affect the relaxivity, including differences in the applied magnetic field, accessibility of water to the particle surface, placement of paramagnetic ions in the UCNPs, and particle size. Among these, UCNP size is an essential aspect to consider when designing multimodal UNCP. Johnson et al.^28^ observed that tiny particles (<10 nm in size) have high molar relaxation values of water molecules owing to the high surface-to-core ion ratio. However, the particle composition should also be considered. It was determined that the exchange of 40% Gd^3+^ with Nd^3+^ in the outer shell of the UCNPs (NaGdF_4_:Yb,Er@NaGdF_4_:Yb,Nd) results in a higher MRI signal and shorter relaxation time. The obtained results indicate that biocompatible core–shell NaGdF_4_:Yb,Er@NaGdF_4_:Yb,Nd UCNPs have shown very good results in cell viability (please refer to Figure 6a) and in the enhancement of the MRI signal intensity. Thus, these novel upconverting nanoparticles can be applied in in vivo MRI measurements as an alternative to the most widely used Gd chelates. However, it is important to note that the adaptation of clinical imaging protocols could lead to an even better MRI performance of the proposed UCNPs.

## 3 Conclusions

In summary, we have demonstrated the synthesis of differently doped (Yb^3+^, Er^3+^, Nd^3+^) core and core–shell NaGdF_4_ upconverting nanoparticles. These nanoparticles can be excited by 808 or 980 nm laser irradiation and exhibit good MRI properties. Moreover, the synthesized nanoparticles exhibited remarkable colloidal stability in a wide range of environments including biological media. Additionally, we determined their temperature-sensing capabilities, including both relative sensitivity (*S*_r_) and absolute sensitivity (*S*_a_). The calculated *S*_r_ and *S*_a_ values at 37 °C (310.15 K) for NaGdF_4_:Yb,Er@NaGdF_4_:Yb,Nd nanoparticles were 1.16% K^–1^ and 2.52 × 10^–3^ K^–1^, respectively. These values demonstrate the effectiveness of the upconverting nanoparticles as temperature sensors in the physiological temperature range. We also evaluated the cytotoxicity of the upconverting nanoparticles using HEK 293T cells, highlighting their potential as safer alternatives to traditional MRI contrast agents, such as Gd-DTPA. The findings of our study encourage further exploration and optimization of upconverting nanoparticles for biomedicine, particularly in the field of molecular imaging, where superb optical/MRI properties and minimizing cytotoxic effects are essential for successful clinical translation.

## Supporting Information Available

Detailed description of synthesis, materials and analysis methods used, as well as additional figures describing the optical characteristics (emission and decay) of presented UCNPs (PDF)

## Abbreviations

DI: deionized
DLS: dynamic light scattering
DMEM: Dulbecco’s modified Eagle's medium
FBS: fetal bovine serum
FIR: fluorescence intensity ratio
FT-IR: Fourier transform infrared
Gd-DTPA: gadolinium diethylenetriamine penta-acetic acid
IEP: isoelectric point
MRI: magnetic resonance imaging
NIR: near-infrared
OA: oleic acid
PL: photoluminescence
PSD: particle size distribution
REF: reference
SEM: scanning electron microscopy
SI: Supporting Information
TEM: transmission electron microscopy
UC: upconversion
UCNPs: upconverting nanoparticles
UV: ultraviolet
Vis: visible
XRD: X-ray diffraction
XTT: sodium 3′-[1-(phenylaminocarbonyl)-3,4-tetrazolium]-bis (4-methoxy6-nitro) benzenesulfonic acid hydrate
