# Supplementary material for: Biocompatible Upconverting Nanoprobes for Dual-Modal Imaging and Temperature Sensing
Source: ACS Appl Nano Mater. 2024 Mar 5;7(6):6185–95. doi: 10.1021/acsanm.3c06111 (PMC10964196; doi:10.1021/acsanm.3c06111)
Supplement: Supplementary file 1 — an3c06111_si_001.pdf [file an3c06111_si_001.pdf]

SUPPORTING INFORMATION

Biocompatible Upconverting Nanoprobes for Dual-Modal Imaging and Temperature Sensing

*Egle Ezerskyte[1,2], Augustas Morkvenas[2,3], Jonas Venius[2], Simas Sakirzanovas[1], Vitalijus Karabanovas[2,3], Arturas Katelnikovas[1], and Vaidas Klimkevicius[1,2]**

[1]Institute of Chemistry, Faculty of Chemistry and Geosciences, Vilnius University, Naugarduko 24, LT-03225 Vilnius, Lithuania

[2]Biomedical Physics Laboratory, National Cancer Institute, Baublio 3b, LT-08406, Vilnius, Lithuania

[3]Department of Chemistry and Bioengineering, Vilnius Gediminas Technical University, Sauletekio 11, LT-10223 Vilnius, Lithuania

vaidas.klimkevicius@chf.vu.lt

1. Materials and synthesis procedures

1.1. Materials

Gadolinium(III) acetate hydrate (99.9%, Alfa Aesar), neodymium(III) acetate hydrate (99.9%, Alfa Aesar), ytterbium(III) acetate hydrate (99.9%, Alfa Aesar), erbium(III) acetate tetrahydrate (99.9%, Alfa Aesar) were dissolved in deionized water (0.2 M) and filtered using 0.2 μm PES syringe filters (ROTH, Chromafil®) before use. Oleic acid (OA, 90% technical grade, Alfa Aesar), 1-ocatadecene (ODE, 90% technical grade, Alfa Aesar) were heated at 120 °C under reduced pressure (15 mbar) for 120 minutes before use. Sodium hydroxide (NaOH, Eurochemicals), ammonium fluoride (NH_4F, 99%, Eurochemicals), HCl solution (HCl, 36.5%, Eurochemicals), methanol (MeOH, HPLC, Eurochemicals), n-hexane (Hex, HPLC, Eurochemicals), cyclohexane (cHex, HPLC, Eurochemicals), diethyl ether (Et_2O, HPLC, Eurochemicals), and acetone (99.8%, Eurochemicals) were used as received unless otherwise specified.

1.2. Synthesis of $NaGdF_4$:Yb^{3+}(18%),Er^{3+}(2%) core upconverting nanoparticles

Freshly prepared aqueous solutions (0.2 M) of gadolinium(III) acetate ($Gd(OAc)_3$, 4 mL, 0.8 mmol), ytterbium(III) acetate ($Yb(OAc)_3$, 0.9 mL, 0.18 mmol), and erbium(III) acetate ($Er(OAc)_3$, 30.1 mL, 0.02 mmol) were poured into a 50 mL three-necked round-bottomed flask and dried to a solid at 90-95 °C. The flask was then cooled to room temperature and a mixture of dry acetates was dispersed in methanol (3 mL) under vigorous stirring. Subsequently, 10 mL of OA and 15 mL of ODE were added to the dispersion of the acetates, and the flask was placed in a heating mantle equipped with a PID temperature controller and a glass-coated thermocouple. The reaction mixture was heated to 140 °C under Ar atmosphere and maintained for at least 40 minutes to remove methanol and any traces of moisture. After cooling down to room temperature, the prepared solution NaOH (1 M in MeOH, 2.5 mL, 2.5 mmol) and NH_4F (0.4 M in MeOH, 10 mL,

4 mmol) were mixed together, shaken for 15 seconds and poured into the reaction mixture. The reaction mixture was then heated to 50 °C and maintained at this temperature for 30 minutes. Afterwards, the temperature was raised to 120 °C to remove methanol from the reaction mixture. When the temperature reached 120 °C, the vacuum line was connected and the reaction solution was mixed at 120 °C under reduced pressure (15 mbar) for an additional 30 minutes. The temperature of the reaction mixture was then raised to 310 °C and maintained for 1 hour. After cooling to room temperature, the reaction mixture was poured into an excess acetone/hexane mixture (4:1 v/v, 150 mL). UCNPs were collected by centrifugation at 10000 rpm for 15 minutes, washed with acetone, acetone/DI water, and again with acetone. It should be noted that the particles were collected by centrifugation after every wash (15 min, 10000 rpm). After the last wash, the collected UCNPs were redispersed in 20 mL cyclohexane and used as a stock solution. The concentrations of the stock solutions were determined gravimetrically.

1.3. Synthesis of $NaGdF_4$:Yb^{3+}(18%),Er^{3+}(2%)@$NaGdF_4$:Yb^{3+}(5%) core-shell upconverting nanoparticles

The required amount of lanthanide acetate solutions: $Gd(OAc)_3$ (0.2 M, 4.75 mL, 0.95 mmol) and $Yb(OAc)_3$ (0.2 M, 0.25 mL, 0.05 mmol) were dried. After adding OA (15 mL) and ODE (22.5 mL), the mixture was heated to 140 °C under Ar atmosphere to remove any traces of moisture. The reaction mixture was cooled to room temperature and 10 mL of the previously synthesized core particles (see Chapter 1.2) was added to the mixture. The cyclohexane was removed using reduced pressure and the prepared solutions of NaOH (1 M in methanol, 2.5 mL, 2.5 mmol) and NH_4F (0.4 M in MeOH, 10 mL, 4 mmol) were mixed together, shaken for 15 seconds and poured into the reaction mixture. The synthesis and purification procedure for obtaining core-shell UCNPs was the same as that for core particle synthesis (see Chapter 1.2.).

1.4. Synthesis of $NaGdF_4:Yb^{3+}$(18%),Er^{3+}(2%)@$NaGdF_4:Yb^{3+}$(5%),Nd^{3+}(40%) core-shell upconverting nanoparticles

The required amount of lanthanide acetate solutions for synthesis: $Gd(OAc)_3$ (0.2 M, 2.75 mL, 0.55 mmol) $Yb(OAc)_3$ (0.2 M, 0.25 mL, 0.05 mmol), and $Nd(OAc)_3$ (0.2 M, 2 mL, 0.4 mmol). The synthesis and purification procedures were identical to those described in Chapter 1.3.

1.5. Oleate ligands removal procedure

5 mL of nanoparticle stock solution in cyclohexane was poured into a 50 mL centrifuge tube, mixed with 4-fold acetone (20 mL), and centrifuged at 12000 rpm for 15 minutes. The collected particles were mixed with deionized water (pH 3-4, adjusted with HCl) and vigorously stirred for additional 3 hours at room temperature. Subsequently, 10 mL of diethyl ether was added and the aqueous/organic solution was mixed. The aqueous phase containing the oleate-free UCNPs was isolated using a separatory funnel. UCNPs were precipitated with acetone (1:3 v/v) and collected by centrifugation at 12000 rpm for 40 minutes. The UCNPs were redispersed in deionized water (pH 3-4), again precipitated with acetone (1:3 v/v), and collected via centrifugation. The UCNPs were then washed with 20 mL acetone and centrifuged again (12000 rpm, 40 minutes). Finally, the collected oleate-ligand-free UCNPs were redispersed in 10 mL of deionized water and stored at room temperature for further experiments. The concentration of the aqueous UCNPs dispersions was determined gravimetrically.

1.6. Cell culturing

HEK 293t human embryonic kidney cells (acquired from Vilnius University) were used for nanoparticle cytotoxicity evaluation. Cells were cultured in a cell growth medium (Dulbecco's Modified Eagle Medium – DMEM), supplemented with 10% fetal bovine serum (FBS), penicillin,

and streptomycin (all from Gibco, USA). Cells were maintained at 37 °C in a humidified atmosphere containing 5% CO_2.

2. Analysis methods

2.1. X-ray diffraction (XRD) measurements

The crystal phase of upconverting core and core-shell nanoparticles (UCNPs) was confirmed by X-ray powder diffraction (XRD) analysis carried on Rigaku MiniFlexII (Rigaku, Japan) diffractometer using Cu Kα radiation.

2.2 Scanning electron microscopy (SEM)

SEM images were taken with a high-resolution scanning electron microscope Hitachi SU-70 (Hitachi, Japan) at an accelerating voltage of 15 kV.

2.3 Transmission electron microscopy (TEM)

Transmission electron microscopy (Philips Tecnai 12 (Philips, USA)) images were used to determine the particle size distribution and morphology of the UCNPs.

2.4 FTIR measurements

FTIR measurements were performed using Frontier FT-IR spectrometer (PerkinElmer). The measurement range and resolution were 550-4000 cm^{-1} and 0.5 cm^{-1}, respectively.

2.5 DLS and zeta potential measurements

The hydrodynamic size distributions of UCNPs were measured by dynamic light scattering (DLS). DLS measurements were carried out on a ZetaSizer Nano ZS (Malvern, UK), equipped with a 4 mW He–Ne laser emitting at a wavelength of 633 nm. Measurements were performed at 25 °C and an angle of 173° using noninvasive backscattering (NIBS) technology. Using NIBS, the particle size detection range was 0.3 nm – 10 µm. The zeta potential values were calculated from

the electrophoretic mobility using the Smoluchowski model at 25 °C. The size and zeta potential distribution data were analyzed using ZetaSizer software from Malvern.

2.6 Cytotoxicity assay

The cells were seeded in 96-wellplate (BD Falcon, USA). After 24 hours, the old medium was replaced with fresh medium containing 10, 20, 50,100 µg/mL UNCPs and a contrast agent. Cell viability was evaluated using a commercially available XTT cell viability assay kit (Molecular Probes, USA). XTT reaction solution was prepared according to the manufacturer's instructions. After incubation, optical density was measured at 490 nm using a microplate reader (BioTek, USA). Absorbance values were calculated as percentage values of viability. The absorbance value of the control group was 100%, and the remaining values were calculated in proportion to the control. Data are presented as the mean ± standard deviation (SD). The statistical significance of the differences between the studied groups was assessed using a two-tailed independent Student's t-test at the 95% confidence level. Statistical significance was set at p-value < 0.05.

2.7 MRI measurements and calculations

2.7.1 T_1 calculation

A clinical 1.5 T MRI scanner (Philips Achieva) was used to evaluate contrast characteristics of UCNPs. Aqueous UCNPs solutions were placed in a series of 1.5 mL plastic Eppendorf test tubes containing solutions of UCNPs and Magnegita® with different concentrations. Images for longitudinal water relaxation time T_1 calculations were acquired using a T_1-weighted (T_1 WI) turbo spin echo inversion recovery sequence. The registration parameters were as follows: time to echo (TE): 10 ms; time to inversion (TI): 20-2000 ms; repetition time (TR): 4000+TI ms; number of

averages: 2; slice thickness: 5 mm; flip angle: 90°; echoes: 1; TSE factor: 8; matrix – 224 × 224; FOV – 120 × 120 mm.

The acquired images were analyzed and the average MR signal intensity (SI) values were obtained by drawing a circular region of interest (ROI) over the test tubes in coronal images using ImageJ software. The ratio, which indicates the number of times the UCNP enhances the signal, was evaluated using Equation (1):

$$\beta = \frac{SI_x}{SI_{H_2O}} \tag{1}$$

SI_x represents the average signal intensity of the UCNPs in the MRI image. SI_{H2O} represents the average signal intensity of water in the MRI image. T_1 was calculated from the images registered under different TI. The signal intensity (SI) values dependent on TI were plotted graphically, and T_1 was calculated by approximating the experimental data by exponential decay using Equation (2):

$$SI(TI) = SI_0\left(1 - 2\left(exp^{-TI/T_1}\right)\right) \tag{2}$$

SI represents the average signal intensity of the UCNPs in the MRI image. SI_0 represents the signal intensity after 1800 inversion pulses were applied.

2.7.2 T_2 calculation

Images for transverse relaxation time T_2 calculation were acquired using a T_2-weighted (T_2 WI) turbo spin echo sequence. The registration parameters were as follows: TE: 10-200 ms; TR: 2500 ms, number of averages: 10; slice thickness: 5 mm; flip angle: 900; echoes: 20; TSE factor: 20. matrix – 224 x 224, FOV – 120 x 120 mm.

T_2 was calculated from images registered under different TE. The SI dependence on TE was plotted graphically, and T_2 was calculated by approximating the experimental data with exponential decay using Equation 3:

$$SI(TI) = SI_0 exp^{-T_E/T_2} \quad (3)$$

The SI represents the average signal intensity of the UCNPs in the MRI image. SI_0 represents the signal intensity after 900 excitation pulses were applied.

2.7.3 Relaxivity r_1 & r_2 calculation

$1/T_1$ and $1/T_2$ values under different Gd molar concentrations has been plotted and approximated linearly. The slope of the line indicates the molar relaxivity values, r_1 and r_2.

3. Supporting figures

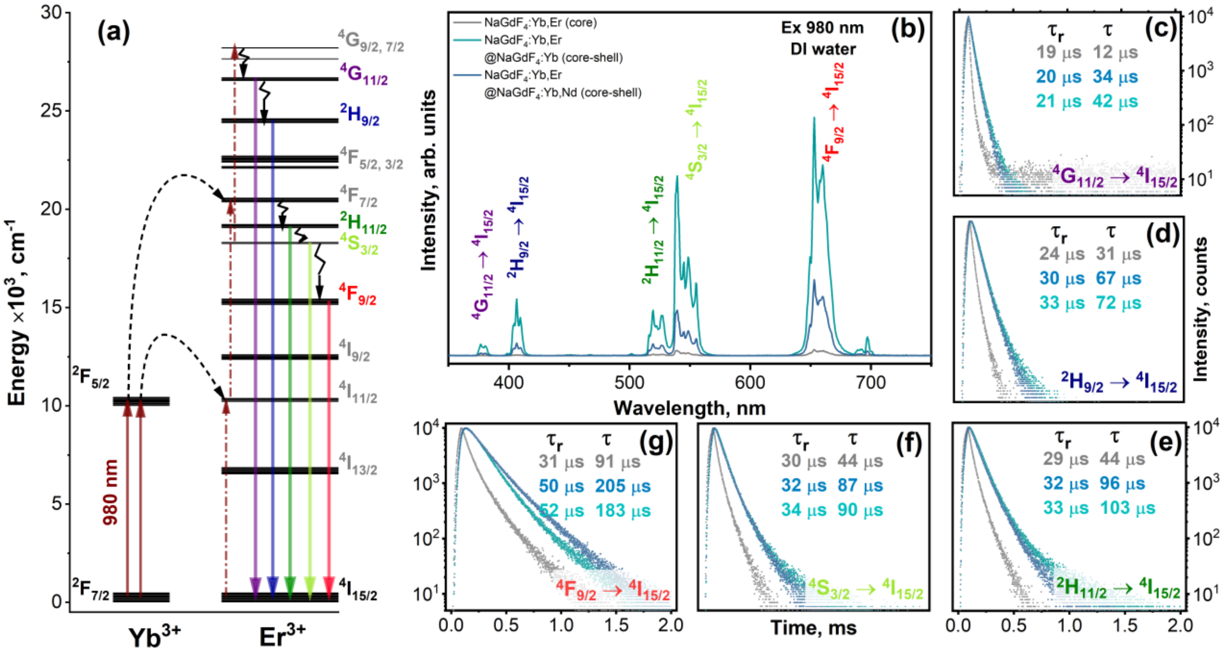

Figure S1. Simplified energy level diagram of Yb^{3+} and Er^{3+} (a); emission spectra of UCNPs (core or core-shell with different composition) dispersed in DI water (b); PL decay curves with calculated UC emission rise time (τ_r) and UC lifetime (τ) values for different emission transitions: $^4G_{11/2}\rightarrow{}^4I_{15/2}$ (c), $^2H_{9/2}\rightarrow{}^4I_{15/2}$ (d), $^2H_{11/2}\rightarrow{}^4I_{15/2}$ (e), $^4S_{3/2}\rightarrow{}^4I_{15/2}$ (f), and $^4F_{9/2}\rightarrow{}^4I_{15/2}$ (g).

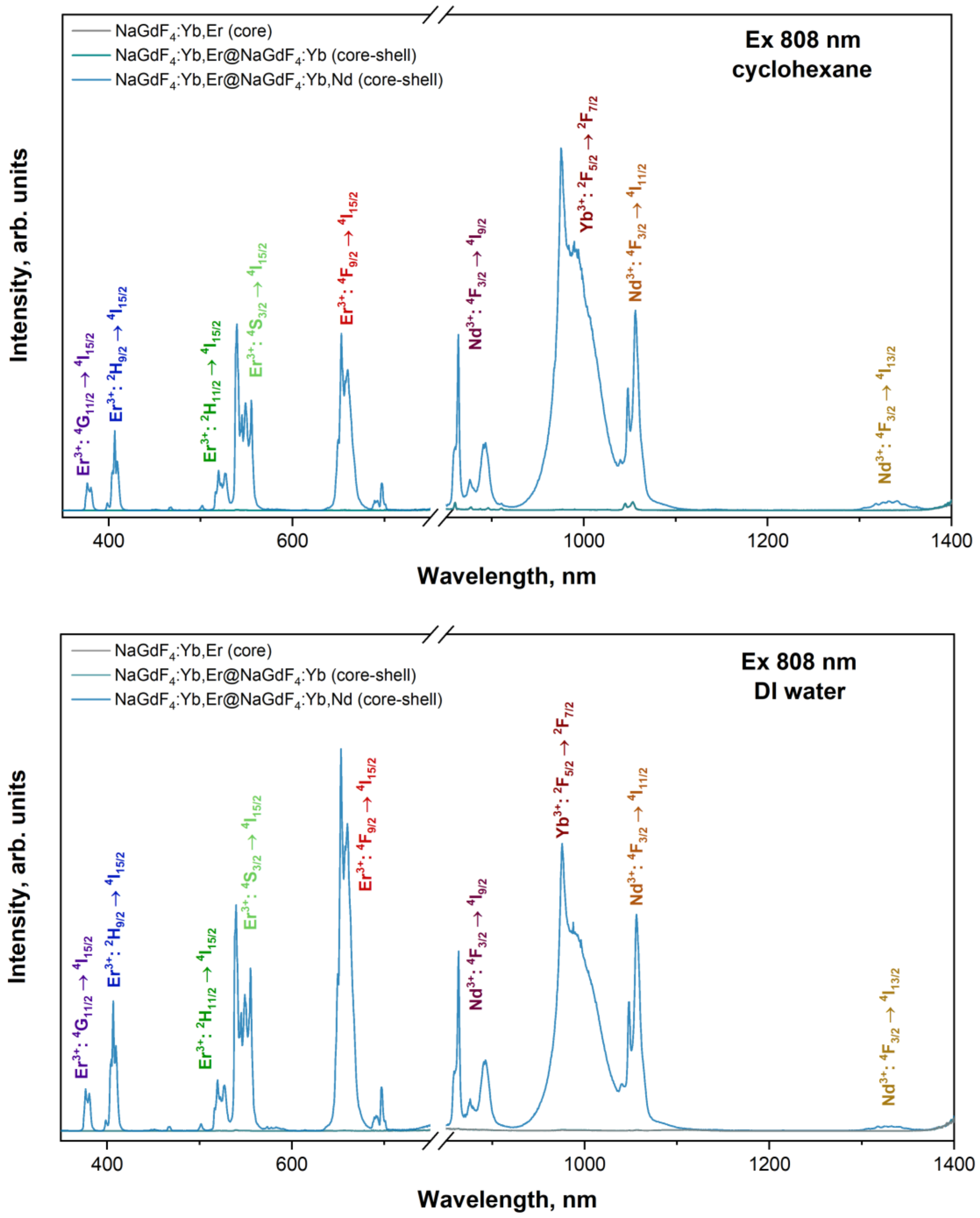

Figure S2. Emission spectra of UCNPs with different architecture (core or core-shell with different composition) measured in cyclohexane (a) and DI water (b) under 808 nm laser excitation.

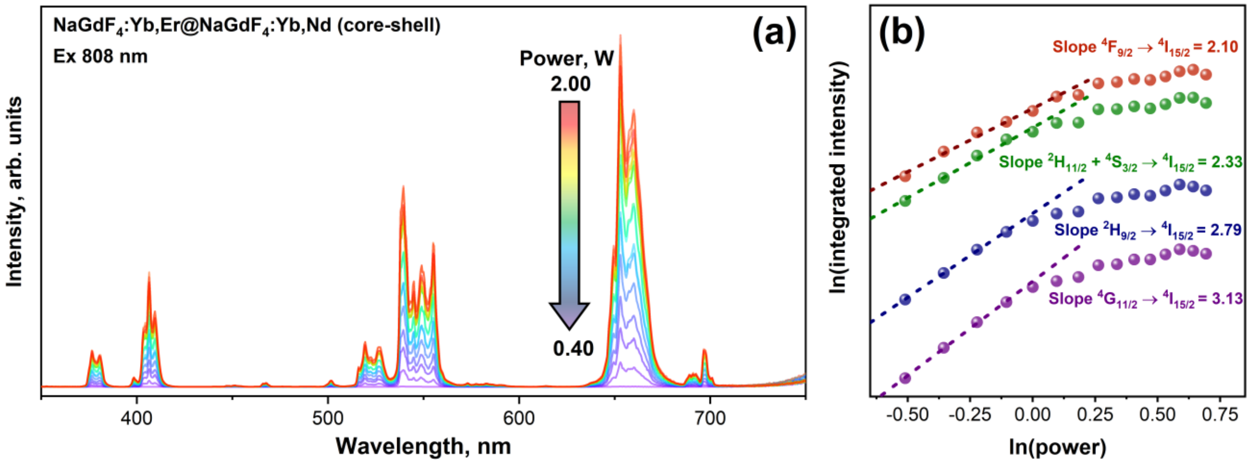

Figure S3. Emission intensity of $NaGdF_4$:Yb,Er@$NaGdF_4$:Yb,Nd UCNPs as a function of 808 nm laser power (a); logarithmic integrated area under the different emission transitions of Er^{3+} as a function of logarithmic power of the laser (b). The slope of linear approximation shows the minimal required number of quants to induce the respective emission transition.

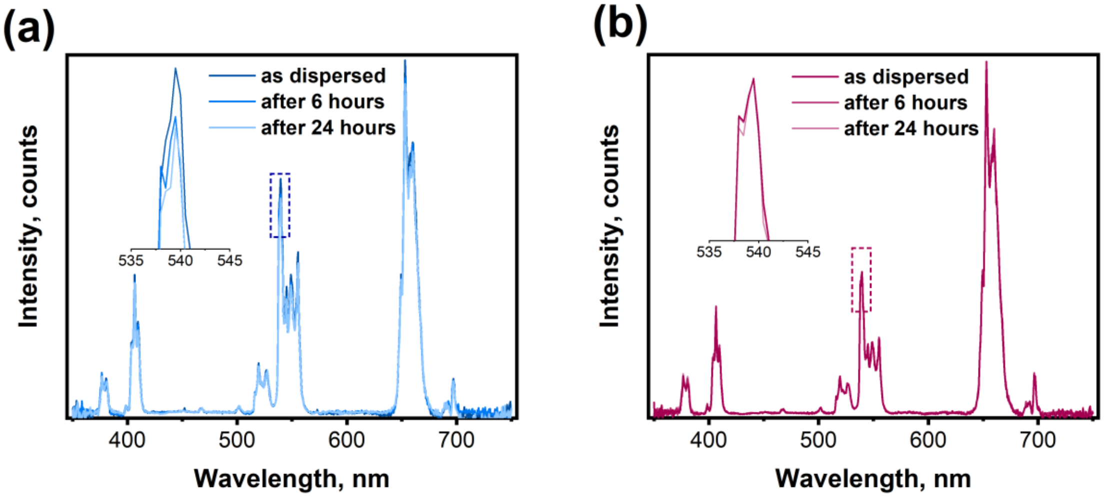

Figure S4. Emission spectra of $NaGdF_4$:Yb,Er@$NaGdF_4$:Yb,Nd core-shell UCNPs recorded in DI water (a) and DMEM+FBS media (b) after a certain duration (as dispersed, after 6 and 24 hours) of stability experiment.

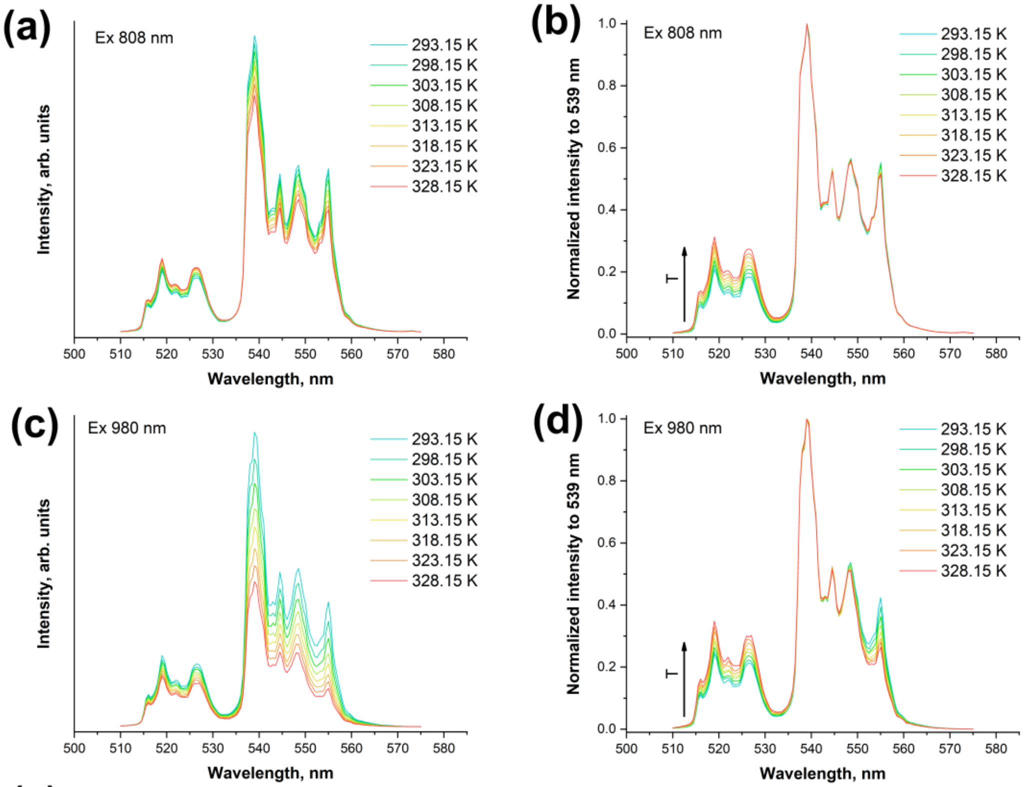

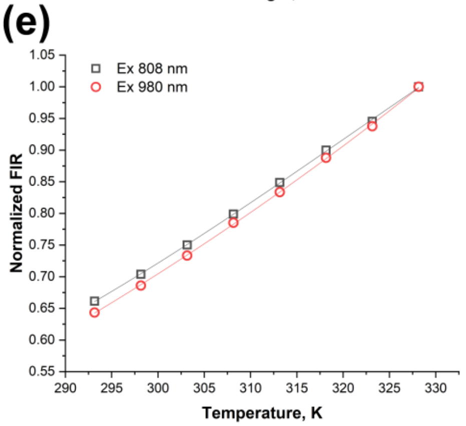

Ex source	T, K	S_r, (%)K^{-1}
$Laser_{808\ nm}$	298.15	1.283
	303.15	1.201
$Laser_{980\ nm}$	298.15	1.362
	303.15	1.275

Figure S5. Temperature-dependent emission intensity (range 510 – 545 nm) (a, c) and normalized (to 539 nm) emission intensity (b, d) of $NaGdF_4$:Yb,Er@$NaGdF_4$:Yb,Nd under excitation with 808 nm (a-b) and 980 nm (c-d) lasers. The normalized fluorescence intensity ratio (FIR) between the integrated areas of the $^2H_{11/2} \rightarrow {}^4I_{15/2}$ and $^4S_{3/2} \rightarrow {}^4I_{15/2}$ energy transitions in Er^{3+} (e) under excitation with 808 nm and 980 nm lasers. The calculated relative sensitivities (S_r) are presented in inserted table.
